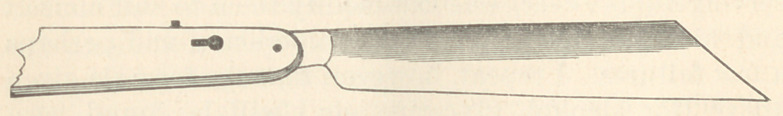# The Microscope in Daily Practice

**Published:** 1876-02

**Authors:** I. N. Danforth

**Affiliations:** Lecturer on Pathology in Rush Medical College, Chicago; 74 South Morgan Street


### THE MICROSCOPE IN DAILY PRACTICE.

(FOURTH PAPER.)

By I. N. DANFORTH, M.D.,

Lecturer on Pathology in Rush Medical College, Chicago.

The solid and semi-solid structures, both healthy and morbid, which require microscopic examination, should be specially prepared for this purpose. Indeed, such preparation is absolutely necessary, if a successful or reliable examination is to be made, or if the operator desires to preserve his specimens permanently. And this leads me to say, that it is entirely within the power of any physician, however busily he may be engaged in practice, to prepare and permanently preserve microscopic specimens of the various tissues, healthy and morbid, until he shall possess a museum of his own, sufficiently extensive for all ordinary purposes of study and comparison. And such specimens are particularly valuable, because the gross appearances of each specimen can be studied, and also because the history, and frequently the ultimate result of each case, can be accurately ascertained, by the possessor of the microscopic sections.

It is vastly important that our younger practitioners be led to understand how much they can do towards building up the science of pathology by carefully and systematically utilizing the material which comes within their reach. One single case, carefully studied and accurately and *conscientiously* reported—especially if death occurs and a post-mortem is obtained—possesses an imperishable value to medical science; and it may be that this single case presents some new feature hitherto unnoticed, which is of vast importance in elucidating some dark point in pathology.

It is really a simple matter to prepare sections of the tissues for microscopic examination, and to preserve them in an unalterable form. But when the process is described on paper, it seems quite complicated. It requires few materials, very little outlay of money, but considerable tact and practice, and inexhaustible patience and industry. It is necessary, also, to do things of this sort in a systematic and workmanlike manner. A care less, unsystematic and slovenly microscopist, is all but —and I think quite—an impossibility. In fact, one of the collateral benefits that any young physician gets out of his microscope, is that through its use he acquires habits of neatness, promptitude and method.

In the present paper I purpose indicating the means necessary for investigating and preserving the histological structures, leaving the methods of their employment for a future paper.

*First.* For the preservation of large specimens, as tumors, or portions of organs which it is desired to keep temporarily or permanently, common commercial alcohol answers every purpose, and is, upon the whole, best of anything. A very valuable preservative fluid, which I have employed for several years, is the following: ℞ Alcohol, Water, *aa* f ℥ x; Glycerine, f ℥ iv; Carbolic Acid, ʒ ij. Mix.

For small specimens, like portions of morbid growths of diseased organs, which are intended for future microscopic study, this fluid is very useful, as it causes much less shrinking and distortion than alcohol. Dr. Keen, of Philadelphia, speaks very highly of solutions of hydrate of chloral, (10 to 20 grains to the fluid ounce of water), as preserving fluids for soft tissues. I have several specimens in chloral solutions, (20 grains to the fluid ounce), and they are keeping very nicely.

It is not necessary to multiply words concerning preserving fluids; every one can modify them to suit himself and his specimens after a little experience, and perhaps a few failures. I repeat, however, that the formula given above (for alcohol, glycerine, etc.) will be found very useful for specimens which it is important should be kept with as little change as possible.

*Second.* Many tissues—for example, the brain and spinal cord, and medullary cancer—must be hardened before microscopic sections can be made. For many specimens, the strongest commercial alcohol answers every purpose; indeed, I rarely find it necessary to employ any other hardening agent. A solution of chromic acid has been much employed, especially by Mr. Lockhart Clarke, for hardening nervous structures. One part of chromic acid is dissolved in 200 parts of water. For hardening portions of brain substance proper, however, the solution must be weaker: say, one part of acid to 300 of water. I have employed this agent considerably, but I think strong alcohol does equally well, and chromic acid is not always at hand, at least in the smaller towns. Other formulæ, for hardening solutions, are given in the text books and journals, but they possess no advantages over those mentioned above, and therefore need not be alluded to.

*Third.* For the satisfactory and profitable examination of the various tissues—both normal and abnormal—very thin slices or sections are requisite, and a variety of instruments have been invented for the purpose of making these sections: such as Valentin's knife, various forms of razors, etc.

Valentin's knife is both complicated and costly, although it is very efficient in skillful hands. An old razor does very well, but is rather too heavy and clumsy for ordinary use. Razor blades, "beveled" upon one side only, are kept by the dealers in microscopists' wares, and are much employed as section knives.

A few years ago, I commenced using the instrument of which the accompanying figure is a fair illustration:

It "consists of a single blade, two and one-half inches long, from heel to point, by three-eighths of an inch in width. It is exceedingly thin, and is, of course, made of the finest steel. It is mounted in a shell handle, is made to close up like an ordinary pocket knife, and is supplied with a pivot, which slides into a slot in the blade to hold it in position, like those usually found in surgical pocket cases. * * * With this simple instrument, I am able to make sections in all respects equal to those I formerly made with Valentin's knife, and with far less trouble." (The "Lens," vol. I, p. 28.) After a larger experience I am prepared to reiterate the opinion I published in January, 1872, concerning the value of this simple and inexpensive instrument for the purpose of making microscopic sections of *all* varieties of soft tissues. Within the past two years, I have procured an improved Valentin's knife, and have also tried my hand at inventing an "improved" razor-shaped knife; but, after a brief experience, I gladly abandoned them both, and returned to the thin-bladed knife just described. And I now recommend it, unhesitatingly, as the best section-instrument with which I am acquainted, because it is inexpensive; because with it any one can quickly learn to make handsome sections, and because they can be made rapidly; because it is easily sharpened, and that without any special dexterity; because it is always ready for use, without any troublesome and delicate adjustment of parts; and, lastly, because it is easily cleaned, and, therefore, easily kept from rusting.

I have dwelt at considerable length upon this matter, in view of the fact—which my own experience has taught me—that an efficient and simple section-knife is a sort of standing temptation to make sections; whereas, a complicated affair has exactly the contrary effect. The knife I recommend is so easy to manage, in all regards, that a man must be the very perfection of clumsiness who can not cut delicate sections after a little experience in its use.

*Fourth.* A few simple implements, involving hardly any cost, like the following, should be provided: A pair of rather coarse, common sewing needles, mounted in wood handles, like the wooden rods employed in making pen-holders; three or four small glass rods, each drawn to a fine point by heating in a spirit lamp: a large-sized, and a few small-sized camel's hair pencils; a dozen of two drachm or half drachm phials fitted with corks; and, of course, a few glass slides and their covers of glass or mica, such as I described in the paper published in the JOURNAL AND EXAMINER for October.

It will be a saving of time and trouble if the above list of articles be provided, before attempting any real work with the microscope. A few reagents will also be necessary. The following are those which are really essential for the physician's ordinary purposes: Alcohol, pure and diluted; sulphuric ether; solution of acetic acid, containing about twenty per cent. of acid; aqueous solution of iodine, made by adding ten or fifteen drops of the "Liquor Iodinii Comp." U. S. P., to an ounce of distilled water; and little slips of both red and blue litmus paper. These reagents should be kept in ounce bottles, with the stoppers so arranged as to allow of the application of a single drop of any one of them to a glass slide. This may easily be done by passing a small glass rod through the cork, and allowing it to project to the bottom of the bottle; a drop or two will always adhere to the rod when it is withdrawn. A very convenient test bottle may be made by passing one of the little instruments called a "medicine dropper," through a cork, in place of the glass rod. The medicine dropper consists of a small rubber bulb, into which is inserted a short pipette; the bulb is allowed to project *above* the cork, while the pipette passes *through* it, and reaches to the bottom of the bottle. If the rubber bulb be compressed, the air is, of course, expelled, and the liquid in the bottle rushes in as soon as the pressure on the bulb is removed; the convenience of this arrangement will suggest itself at once. Glass-stoppered bottles, with the stoppers drawn out into delicate glass rods, reaching nearly to the bottom of the bottle, may be obtained of the dealers in chemical wares, but they are no better than the "home-made" contrivances already described. Dealers in dentists' supplies also keep bottles in which the upper portion (the "handle") of the glass stopper is a hollow bulb, while the lower portion is a small glass rod reaching almost to the bottom of the bottle. If the bulb be gently heated in the flame of a spirit lamp, or in hot water, the air is mostly expelled; if, now, the open extremity of the rod be plunged beneath a liquid, the latter will mount in the glass tube and fill the bulb more or less completely; if the bulb be held between the fingers, it will always be sufficient to expel a drop or two of the reagent. These bottles cost twenty-five cents each, and they are the most convenient of any yet devised.

The length to which this paper has already reached, admonishes me that I shall be compelled to defer what I have to say upon staining fluids, preservative media, etc., until a future time.

74 South Morgan Street.

## Figures and Tables

**Figure f1:**